# Design and rationale for Exploring Predictors of successful healthCare Transition for adolescents with chronic conditions in a longitudinal explorative cohort

**DOI:** 10.1016/j.hctj.2026.100129

**Published:** 2026-02-26

**Authors:** M.S. Vletter, J.W. Hoefnagels, A.L. van Staa, S.L. Nijhof, J.W. Gorter, J.N.T. Sattoe

**Affiliations:** a Research Centre Innovations in Care, Rotterdam University of Applied Sciences, Rotterdam, the Netherlands; b Department of Paediatrics, Wilhelmina Children's Hospital, University Medical Centre Utrecht, Utrecht University, Utrecht, the Netherlands; c Department of Rehabilitation, Physical Therapy Science & Sports, University Medical Center Utrecht, Wilhelmina Children's Hospital, Utrecht, the Netherlands; d Center of Excellence for Rehabilitation Medicine, UMC Utrecht Brain Center, University Medical Center Utrecht, and De Hoogstraat Rehabilitation, Utrecht, the Netherlands; e CanChild, Department of Pediatrics, McMaster University, Hamilton, Ontario, Canada

**Keywords:** Transitional care, Transition to adult care, Biopsychosocial model, Adolescent health, Chronic disease

## Abstract

**Background:**

Transitioning from paediatric to adult healthcare is a pivotal phase for adolescents and young adults (AYAs) with chronic conditions. Effective healthcare transition (HCT) supports continued healthcare engagement and stable psychosocial outcomes, reducing risks for patients, their families, and the healthcare system. Yet the determinants of a smooth transition are complex, shaped by the interplay of various biopsychosocial constructs.

**Objective:**

The ExPeCT study (acronym: Exploring Predictors of a successful healthCare Transition) seeks to: A) examine the relationship between biopsychosocial development, transition readiness, and HCT outcomes, including patients’ experiences; and B) identify key predictors of successful HCT in AYAs with chronic conditions.

**Method:**

This longitudinal, observational, exploratory cohort study is embedded in the PROactive cohort, which annually measures biopsychosocial well-being of children with chronic conditions. The ExPeCT study supplements the PROactive cohort by collecting additional data on HCT experiences at three time points: once in paediatric care and twice after transfer to adult care.

Data will be obtained from a range of chronic disease groups. Advanced statistical modelling will be employed to analyse transition outcomes and experiences, and to investigate associations between biopsychosocial development and transition readiness.

**Significance:**

This study is expected to fill a critical gap in the field by providing comprehensive understanding of predictors of successful healthcare transition in AYAs with chronic conditions. The insights gained will help improve care strategies and guide healthcare professionals in supporting successful transition to adult care.

## Highlights

• Predictors for a successful healthcare transition are explored in a unique longitudinal dataset during healthcare transition.
• The longitudinal dataset includes patients with multiple chronic conditions undergoing various transition protocols.
• Findings will offer concrete targets for designing or improving transition interventions.

## 1 Introduction

Improved medical care has enabled longer lifespans for children with chronic conditions – even up to and beyond adulthood.^1^, ^2^ However, as they come of age, they face recurring challenges related to education, career choices, intimate relationships, and changes in living situations. On top of these universal developmental challenges, adolescents and young adults (AYAs) with chronic conditions must also navigate through a critical juncture in their healthcare journey: the transition from paediatric to adult care.^3^

Blum et al. defined this transition as *‘the purposeful, planned process that addresses the medical, psychosocial, educational and vocational needs of adolescents and young adults …as they move from child-centred to adult-oriented healthcare systems’.*^4^ The transition process requires adjustment to a new care environment, and more responsibility for managing one’s own health. Moreover, the AYAs need to cope with both the psychological and social consequences of growing up and moving into adult healthcare.^5^

Healthcare transition (HCT) is a complex, multifaceted process with considerable risks for various stakeholders when poorly managed.^6^, ^7^ Poor HCT outcomes can result in higher long-term financial and time burdens for the healthcare system.^8^ Families of transitioning patients may experience stress from letting their child go, often describing the sensation of “falling off the cliff”.^7^ For patients themselves, poor healthcare management during the HCT can lead to missed appointments, decreased medication adherence, worsening disease activity, and increased symptomatology.^9^ Psychosocially, AYAs with chronic conditions may experience lower self-efficacy, reduced independence, diminished empowerment, and atypical social participation – all negatively impacting quality of life.^10^, ^11^ Although the HCT process may play a role in these negative outcomes, these outcomes can, in turn, negatively influence HCT itself.^12^ The various factors that interact with HCT outcomes can be categorized within the biopsychosocial model, which advocates a holistic view of health by integrating biological, psychological, and social dimensions.^13^ Some of these factors – for instance, transition readiness – overlap multiple dimensions of the biopsychosocial model.

Transition readiness is the preparedness of AYAs to manage the transfer from paediatric to adult healthcare.^2^, ^14^ The Social-ecological Model of AYA Readiness for Transition (SMART) distinguishes between modifiable (subjective) and non-modifiable (objective) factors that influence the transition readiness of AYAs.^15^ The modifiable factors are potential targets of transition interventions, including knowledge about the disease, health status and transition; healthcare management skills/efficacy; personal beliefs, expectations, and goals for the transition process; and psychosocial functioning. For these factors, both barriers and facilitators, as well as the perspectives of multiple stakeholders, are considered, making this model a useful framework for identifying focus points for transition interventions. Consequently, many HCT interventions aim to improve transition readiness as a pathway to more successful transitions.^16^ The availability of longitudinal data, which so far are scarce, could shed light on the relation between interventions and factors like transition readiness and successful HCTs.

Moreover, there is still no consensus on what constitutes a successful HCT. Healthcare providers mainly focus on the continuation of care and treatment adherence as key indicators.^17^ From parents' and AYAs’ perspectives, a successful HCT involves a gradual process, supervised by the paediatric health professional, towards independence in adult care.^18^ Nguyen and Cury broadened the perspective by highlighting domains such as healthcare, housing, and finances, and pointing out that adequate life skills such as resilience, leadership, and education are needed to navigate adult life.^19^ Themes for a successful transition demonstrated by the Delphi study of Fair et al. include: knowledge and skills, psychosocial well-being, and self-management/disease control.^20^ All stakeholder perspectives are relevant for transition success, including AYAs satisfaction with the HCT process and their need for a more holistic approach with attention to psychosocial factors.^5^, ^17^, ^21^, ^22^ These perspectives are integrated in the definition of a successful HCT of Toulany et al.: *“A successful transition ensures care that is continuous, coordinated, and adapted to each youth’s development and maturity, while improving (or at least maintaining) disease control, patient satisfaction, quality of life, and social participation throughout young adulthood”.*^23^ Following this broad definition, in this study successful HCT will be operationalized as a composite of biological, psychological, and social construct, and healthcare use.

The cross-sectional biopsychosocial factors that affect transition readiness, experiences, and outcomes have been well established. Still, longitudinal data remain scarce.^2^ Van Staa et al. evaluated the HCT experiences of AYAs six years after the event, but did not assess longitudinal changes in biopsychosocial functioning, except for quality of life.^21^ Colver et al. annually tracked the biopsychosocial development of AYAs with type 1 diabetes, cerebral palsy, or autism spectrum disorder after HCT.^24^ Yet, this study primarily focused on AYA’s satisfaction with services, wellbeing, and participation, rather than on process indicators like attendance or loss to follow-up.^24^ Moreover, transition readiness was assessed in neither of the studies. This observation highlights the need for a comprehensive, longitudinal approach that integrates biopsychosocial development, transition readiness, experiences, and outcomes.

To address this knowledge gap, the longitudinal ExPeCT study (acronym: Exploring Predictors of a successful healthCare Transition) will involve an observational explorative cohort. Specifically, the study aims to A) examine the relationship between biopsychosocial development, transition readiness, and HCT outcomes, including AYAs’ experiences; and B) identify key predictors of successful HCT in AYAs with chronic conditions. We hypothesize that greater transition readiness, and higher mental and social functioning will be associated with more favourable HCT outcomes in adult care. The ExPeCT study is embedded in an existing biopsychosocial longitudinal cohort in paediatric care in the Netherlands. Findings from this study will help inform future care strategies and promote a more integrated, person-centred approach to healthcare transition.

## 2 Methods

### 2.1 Study design

**Fig. 1:**
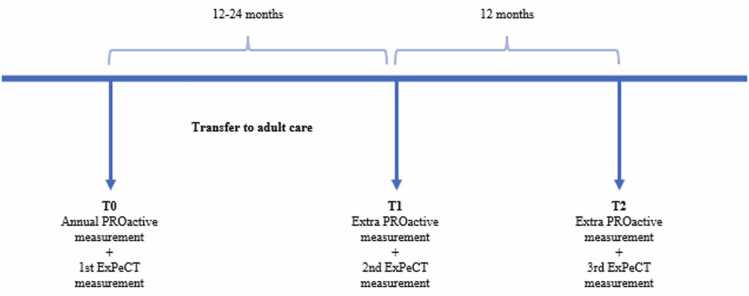
Overview of measurement moments. The time interval between T0 and T1 depends on the exact timing of transfer.

The ExPeCT study employs a combined retrospective-prospective quantitative cohort design within a single group of participants. It builds on the ongoing, care-based PROactive cohort study, which uses established methods to assess psychosocial wellbeing in children with chronic conditions.^25^ Since 2016, the PROactive cohort study has annually followed an expanding number of children with chronic conditions receiving outpatient care at the Wilhelmina Children’s Hospital in Utrecht, the Netherlands, and currently includes data from more than 3500 patients. The ExPeCT study uses data from the PROactive study database. So in retrospect, the ExPeCT study is provided with a rich longitudinal biopsychosocial baseline, capturing biopsychosocial developmental trajectories through age 18. Prospectively, the ExPeCT study extends the PROactive cohort by collecting additional data on transition outcomes and biopsychosocial development before and after AYAs’ transfer to adult care. Participation in the ExPeCT study begins during one of the final PROactive measurements in paediatric care and comprises three measurement moments: one in paediatric care and two follow-up assessments in adult care. The exact timing of the measurements and the overall duration of participation vary depending on the individual’s transfer date (see Fig. 1).

### 2.2 Participants

Participants are recruited from disease groups in the PROactive cohort study, including chronic kidney disease, autoinflammatory conditions such as juvenile idiopathic arthritis (JIA), inflammatory bowel disease, and congenital heart disease. Inclusion criteria are AYAs with a chronic condition who are 16–18 years old at the time of inclusion, have completed the PROactive baseline assessment, and have at least one remaining paediatric PROactive assessment. All eligible AYAs are invited to participate, regardless of whether they are transferring internally within the University Medical Centre Utrecht (UMCU) or externally to another medical centre. In 2024, 227 PROactive participants were aged 16–18. Additional participants are approached as they become eligible.

### 2.3 Procedures

Inclusion for the ExPeCT study began in October 2024. PROactive participants are screened for eligibility, and healthcare professionals initially assesses their willingness to participate. Interested AYAs are then contacted by a research member, provided with information, and sent a patient information letter. Upon consent, the first ExPeCT assessment (T0) coincides with the next annual PROactive measurement. The second assessment (T1) occurs 12–24 months later, depending on transfer to adult care; for those transferring externally, T1 is aligned with the last paediatric consultation and PROactive measurement. The final assessment (T2) is conducted one year after the first adult care measurement (T1), with participants typically aged 19–20 at study completion.

### 2.4 Measurements

Data collection in the PROactive cohort originally involves questionnaires and chart reviews. A complete overview of the PROactive questionnaires, including psychometrics and changes over time, is available on the DataverseNL page of the PROactive cohort study.^26^

In the ExPeCT study, PROactive data collection is continued beyond the transfer to adult care and expanded to include questionnaires addressing psychosocial aspects of HCT and AYAs’ HCT experiences, as well as chart reviews capturing HCT outcomes.

Table 1 provides an overview of all measured constructs. A detailed description of these constructs follows, organized by category: biological, psychological, social, and healthcare use. Measurement tools added specifically for the ExPeCT study are described in more detail.

**Table 1:** Overview of outcome measures!.

**Domain**	**Outcome**	**Measurement instrument**	**Measurement moments**
**Biological**	General (gender, age)	PROactive General Questionnaire^a^, ^b^	T0-T1-T2
	Somatic diagnosis Comorbidities Disease duration Disease activity^c^ Hospital admission	Chart review	
	Fatigue	• Checklist Individual Strength - 4^27^ • PROMIS Paediatric Fatigue v2.0 Short Form 4a • Numeric Rating Scale - Fatigue 1a^28^	
	Sleep disorder	• Numeric Rating Scale - Sleep 1a^29^ • PROMIS Paediatric Sleep Disorders v1.0 Short Form 4a^30^	
	Physical functioning	• PROMIS Paediatric Mobility v2.0 Short Form 4a^30^ • PedsQL- Generic Core Scales v4.0 Short Form 15 a^,^ ^31^ • PROactive General Questionnaire^a^^,^^b^	
	Pain	Numeric Rating Scale - Pain 1a^32^	

**Psychological**	General	• Numeric Rating Scale - Experienced Health 1a^32^ • PROactive General Questionnaire^a^^,^^b^	T0-T1-T2
	Emotional functioning	PedsQL-Generic Core Scales v4.0 Short Form 15 a^,^^31^	
	Mental functioning	• Revised Children’s Anxiety and Depression Scale^33^ • PROMIS Anger v2.0 Short Form^30^	
	Self-efficacy*	• General Self-Efficacy Scale^34^ • Resilience Scale for Adolescents a^,^ ^35^	
	Trust in healthcare providers*	Visual Analogue Scale 1–10^36^	

**Social**	General: Family composition, conditions, general health	PROactive General Questionnaire^a^^,^^b^	T0-T1-T2
	Quality of life	PedsQL Generic Core Scales^31^	
	School/work participation	• PROactive School Participation^b^ • PROactive General Questionnaire^a^^,^^b^ • PedsQL- Generic Core Scales v4.0 SF15^a^^,^ ^31^	
	Socioeconomic status	PROactive General Questionnaire^a^^,^^b^	
	Social functioning (family/peers)	• PROMIS Paediatric Relationships with Peers v2.0 Short Form 4a^30^ • PedsQL- Generic Core Scales v4.0 Short Form 15a,^31^ • PROactive General Questionnaire^a^^,^^b^ • Resilience Scale for Adolescents^a^^,^	
	Empowerment*	Gothenburg Young Persons Empowerment Scale^11^	
	Transition to adulthood*	Rotterdam Transition Profile^37^	
	Transition readiness*	Transition Readiness Assessment Questionnaire-6.0^38^	T0-T1
	Transition experiences*	• On Your Own Feet – Transition Experience Scale^21^ • Visual Analogue Scale 1–10 - Satisfaction^36^	T1

**Healthcare use**	Date of transfer	Questionnaire & chart review^d^	T0-T1-T2
	Last appointment in paediatric care		
	First appointment in adult care		
	Number of scheduled consultations per year		
	Number of telephone consultations per year		
	Number of missed consultations per year		
	Number of emergency department (ED) visits per year *In case: Reason for ED visit*		
	Number of disease-related hospital admissions per year* In case: Reason for hospitalisation		

! Background and additional information on PROactive questionnaires are discussed elsewhere.

a The questionnaire is multidimensional, assessing multiple constructs.

b Based on the Health Behaviour in School-aged Children (HBSC) questionnaire.^39^

c The exact outcome measures for disease activity per patient group will be published on DataverseNL.^26^

d Measurement tools added for constructs of the ExPeCT study are described in more detail in the text.

* Outcomes are added to the PROactive cohort for ExPeCT study.^25^

#### 2.4.1 Biological

Biological constructs measured via PROactive questionnaires are fatigue, sleep disorders, pain, and physical functioning. Additionally, information on disease activity is collected through chart review. The outcomes vary per disease group.^25^ The ExPeCT study does not add new biological constructs.

#### 2.4.2 Psychological

Psychological constructs in PROactive questionnaires are life events, emotional functioning, anxiety, depression, and resilience. The ExPeCT study adds empowerment and self-efficacy.

Empowerment is measured with the Gothenburg Young Persons Empowerment Scale (GYPES), which includes 15 items, three for each of the dimensions identity, knowledge and understanding, personal control, decision-making, and enabling others.^11^ The responses to the items follow a five-point Likert scale: strongly disagree, disagree, neither agree nor disagree, agree, and strongly agree. A higher score indicates a higher level of patient empowerment (α = 0.858).^11^

The General Self-Efficacy Scale (GSES) is a 10-item measure of an individual’s ability to reach goals and accomplish a variety of tasks.^40^ Items are rated on a 4-point scale, ranging from ‘not at all true’ (1) to ‘exactly true’ (4). Scores are summed to obtain a total general self-efficacy score, with higher scores indicating a greater sense of perceived self-efficacy (α = 0.76–0.89).^34^

#### 2.4.3 Social

PROactive questionnaires assess eight social constructs: level of education, socioeconomic status, family composition, family members with a chronic condition, social functioning, social support, school/work pressure, and family empowerment. For the ExPeCT study, additional measures include social participation, transition readiness, and transition experiences.

Social participation is measured using the validated Rotterdam Transition Profile (RTP).^37^ The RTP comprises nine items assessing AYAs current situation regarding education and employment, finances, housing, transportation, romantic relationships, leisure, health care needs, services and aids, and condition-specific specialist services.^37^ Each item corresponds to one of three phases in the transition to adulthood, with higher scores indicating greater social participation in adult activities (α = 0.82 in Portuguese translation).^41^

Transition readiness is measured with the Dutch translation of the Transition Readiness Assessment Questionnaire (TRAQ-6.0) (α=0.87) before and after the transfer to adult care.^38^ The TRAQ is a 20-item self-report measure assessing four domains: appointment keeping, tracking health issues, managing medications, and communicating with providers.^38^ Two additional items measure the importance of self-management and patients’ trust in their own skills in relation to healthcare management. Responses are scored on a five-point scale based on the Transtheoretical Stages of Change Model. Scores range from ‘I do not know how to do this’ or ‘not at all important’ (lowest readiness - 1) to ‘I always do this when I need to’ or ‘very important’ (highest readiness -5).^42^ The TRAQ has been widely used to measure changes in transition readiness over time and to evaluate HCT experiences in AYAs receiving adult care.^38^

The transition experiences of AYAs are measured with the On Your Own Feet-Transition Experience Scale (OYOF-TES).^21^ Its 20 items are rated on a 5-point Likert scale (1 = strongly disagree to 5 = strongly agree). Three Visual Analogue Scales from 1 to 10 evaluate satisfaction with the HCT process and trust in both paediatric and adult healthcare providers, with higher scores indicating more positive experiences (α = 0.93).^36^

#### 2.4.4 Healthcare use

The biological chart review of the PROactive cohort is expanded to include constructs such as adherence to medical consultations, hospitalizations, and transfer date.

This data collection is continued into adult care for AYAs according to the scheduled measurement moments. For those transitioning to other medical centres, chart data after transfer are unavailable; therefore, an additional questionnaire covering these topics is administered at T1 and T2. This questionnaire is also provided to AYAs at UMCU, allowing their responses to be cross-checked against chart data.

Since each disease group employs different transition activities, this may result in varying effects on transition experiences across disease groups. Information on the use of the core interventions as indicated in the Dutch Quality Standard on transitional care was inventoried at the start of the project and followed up on to allow adjustments in the analysis.^43^

### 2.5 Data management

The ExPeCT study is embedded in the PROactive study, which has an approved data management plan adhering to the FAIR principles -Findable, Accessible, Interoperable, and Reusable – for its data.^44^ Although the data cannot be publicly shared due to their sensitive nature, ExPeCT data are findable within the PROactive cohort via a DOI on DataverseNL.^26^ Available information includes the data management plan, a description of the data, and a Data Access Protocol outlining the procedures and guidelines for requesting and reusing the data.

### 2.6 Ethical considerations

Digital informed consent is obtained from AYAs at T0, in accordance with the Dutch Medical Treatments Contracts Act (WGBO) and the Central Committee on Research Involving Human Subjects (CCMO) guidelines that waive parental consent from age 16.^45^, ^46^ To promote retention, participants receive a total compensation of €50. This is divided into three gift vouchers provided after completion of each measurement (€15, €15, €20). This compensation complies with the CCMO guidelines.^45^ The PROactive cohort study complies with European data protection and privacy regulations. The Medical Ethics Review Committee of UMC Utrecht determined that the study does not fall under the scope of the Medical Research Involving Human Subjects Act (non-WMO; 16-707/C and 17-078/C). The ExPeCT study is covered under the PROactive amendment 24U-0114 (2024).

### 2.7 Data analysis

Descriptive statistics will be applied to describe the study population. Given the extensiveness of the measurements, two types of missing data are anticipated. The first arises when participants end a measurement early or skip items or questionnaires; multiple imputation will be used to address this. The maximum missing data rate is set at 50 % of the total number of outcomes per ExPeCT assessment, to account for the limited effectiveness of multiple imputation.^47^ If more data are missing, the data of that assessment will be excluded from the analysis. If two out of the three ExPeCT assessments are unavailable, the participant’s entire dataset will be excluded from the analysis. The second type of missing data involves missing annual measurements in the PROactive database, as not all participants completed every annual assessment and baseline measurement were conducted at varying ages, resulting in different numbers of follow-ups between participants. Generalized Estimating Equations (GEE) and linear mixed models (LMM) will be employed to account for repeated measures and variability in measurement timing. These methods also accommodate constructs added specifically for the ExPeCT study, such as transition readiness (assessed at two time points) and transition experiences (assessed once).

Predictors for a successful transition will be identified using cluster analysis. First, patterns in biopsychosocial development will be identified. Data from the PROactive database are combined with data of the ExPeCT study, followed by an exploratory hierarchical cluster analysis using the complete linkage method with a simple matching coefficient to derive the number of patterns resulting from the data. A two-step cluster analysis will follow to distinguish patterns on successful transition experiences and outcomes. ANOVA will be used to analyse the effect of different transition interventions on outcomes, and ANCOVA will be used to adjust for these effects. Overall, within-group changes across measurement points and predictors of successful transition will be analysed using multivariate analysis of covariance, while correlations will be assessed using Pearson's or Spearman's tests, as appropriate.

The threshold for statistical significance is two-tailed P-value < 0.05. Statistical analyses will be performed using R statistical software (R Foundation for Statistical Computing, Vienna, Austria).

## 3 Strengths and limitations

The ExPeCT study will explore the development and interplay of biopsychosocial factors, transition readiness, and transition outcomes, aiming to identify predictors of successful HCT in AYAs with chronic conditions. By collecting comprehensive data across biological, psychological, social, and healthcare-use domains, the study provides a unique contribution to understanding the psychosocial well-being of AYAs during the transition from paediatric to adult care.

When considering biological factors, it is important to note that AYAs with lower disease activity tend to exhibit lower transition readiness levels, which may contribute to less successful healthcare transitions.^48^ Since participants in this study are recruited from an academic medical centre, higher baseline disease activity is anticipated, potentially influencing baseline transition readiness.^49^ Conversely, in clinically stable patients, disease activity during HCT may be minimal, with follow-up limited to biennial consultations, which could also result in lower transition readiness. Biological factors are generally less modifiable by short-term transition interventions, and their effects on transition outcomes cannot be adjusted. Accordingly, in this study, biological factors are treated as non-modifiable variables, consistent with the SMART model.

It is essential to acknowledge the variability inherent in the data due to the study’s embedding within the care-based PROactive cohort. Differences may arise in the number of measurement moments, timing of intervals, and availability of chart data for AYAs transferring to external institutions. While this variability adds complexity to the analysis, it also allows for examining the long-term trajectories of biopsychosocial factors and their association with diverse HCT experiences. Advanced statistical methods, such as longitudinal mixed models, will be employed to account for these differences and ensure robust analysis.^50^ However, the development of AYAs over a year may not be fully captured by the selected questionnaires, and observed changes may be influenced by external factors beyond the study’s scope of this study. These observations will be considered when interpreting the findings.

Given the exploratory character of the study, the accuracy and selection of measurement tools are essential. Recent literature on relevant constructs for HCT guided questionnaire selection, and all questionnaires used are validated. For clarity, constructs are organised into four categories: biological, psychological, social, and healthcare use, although some constructs span multiple categories. This will be considered when reporting results. The cluster analysis will provide valuable insights into patterns in the data. However, no reference data on biopsychosocial development in healthy peers are included.

Another challenge concerns potential selection bias. Not all patients treated at the Wilhelmina Children’s Hospital are willing to participate in the PROactive cohort, and among those who do, not all eligible AYAs may opt to join the ExPeCT study. In addition, not all participants will complete every measurement point. Given that AYAs undergo substantial developmental changes during this life phase, loss to follow-up may occur due to declining interest or competing priorities.^37^ Consequently, completed participation in the ExPeCT study may reflect active engagement with one’s healthcare path. As a result, the final participant sample may display higher-than-average transition-readiness scores.^51^ Although this bias is difficult to eliminate in voluntary longitudinal cohort studies, it will be addressed in the analysis, and the findings will be interpreted in the context of existing literature on transition readiness.^51^, ^52^

A strength of the ExPeCT study is the inclusion of a heterogeneous patient population who will not only transfer internally but also externally to various other adult care settings. This diversity in participants will enhance the generalizability of the results. Nevertheless, potential variability in the HCT protocols of different disease groups may weaken this generalizability across the cohort and limit the comparability between groups. However, it also offers an opportunity to evaluate the influence of different HCT protocols on transition outcomes. Comparing the outcomes of these protocols will yield insights into the best practices in supporting AYAs towards a successful HCT.

Ultimately, analysis of the ExPeCT longitudinal dataset will help predict successful HCT experiences among AYAs with chronic conditions. Understanding how biopsychosocial factors develop during HCT and how these interact with transition readiness, may inform more individualised and holistic transition strategies. This knowledge could improve healthcare outcomes, support AYAs’ psychological and social well-being, and assist healthcare providers in developing sustainable clinical guidelines for the HCT. Additionally, the results will contribute to defining what constitutes a successful transition, guiding the development of policies aimed at standardising and optimising transition protocols for AYAs with chronic conditions.

## CRediT authorship contribution statement

**Sanne L. Nijhof:** Writing – review & editing, Methodology, Funding acquisition, Conceptualization. **AnneLoes van Staa:** Writing – review & editing, Methodology, Funding acquisition, Conceptualization. **Johanna W. Hoefnagels:** Writing – review & editing, Supervision, Resources, Methodology, Funding acquisition, Conceptualization. **Maartje Sophia Vletter:** Writing – review & editing, Writing – original draft, Methodology. **Jane N.T. Sattoe:** Writing – review & editing, Supervision, Project administration, Methodology, Funding acquisition, Conceptualization. **Jan Willem Gorter:** Writing – review & editing, Methodology, Conceptualization.

## Ethical

Consent and approval from the University of Utah Institutional Review Board (IRB) was completed prior to initiation of this research (IRB_00115964). This research was conducted in accordance with Helsinki Declaration as revised in 2013. Written informed consent was obtained from the participants of the study.

## Funding

This study protocol is part of the ‘Transition Continued’ Project, supported by funding from the Taskforce for Applied Research SIA of the Dutch Research Council (Grant no.: RAAK.PRO05.057).

## Declaration of Competing Interest

Dr. A.L. van Staa is the guest editor for this special issue and also a co-author of this paper. The other authors report no conflicts of interest.

## Data Availability

No data was used for the research described in the article.
